# Effects of combined antiretroviral therapy on B- and T-cell release from production sites in long-term treated HIV-1^+^ patients

**DOI:** 10.1186/1479-5876-10-94

**Published:** 2012-05-16

**Authors:** Eugenia Quiros-Roldan, Federico Serana, Marco Chiarini, Cinzia Zanotti, Alessandra Sottini, Daria Gotti, Carlo Torti, Luigi Caimi, Luisa Imberti

**Affiliations:** 1 Institute of Infectious and Tropical Diseases, University of Brescia, Brescia, Italy; 2 Laboratory of Biotechnology, Diagnostics Department, Spedali Civili of Brescia, Brescia, Italy

**Keywords:** KRECs, TRECs, HIV-1, cART, T lymphocytes, B lymphocytes

## Abstract

**Background:**

The immune system reconstitution in HIV-1- infected patients undergoing combined antiretroviral therapy is routinely evaluated by T-cell phenotyping, even though the infection also impairs the B-cell mediated immunity. To find new laboratory markers of therapy effectiveness, both B- and T- immune recovery were evaluated by means of a follow-up study of long-term treated HIV-1- infected patients, with a special focus on the measure of new B- and T-lymphocyte production.

**Methods:**

A longitudinal analysis was performed in samples obtained from HIV-1-infected patients before therapy beginning and after 6, 12, and 72 months with a duplex real-time PCR allowing the detection of K-deleting recombination excision circles (KRECs) and T-cell receptor excision circles (TRECs), as measures of bone-marrow and thymic output, respectively. A cross sectional analysis was performed to detect B- and T-cell subsets by flow cytometry in samples obtained at the end of the follow-up, which were compared to those of untreated HIV-1-infected patients and uninfected controls.

**Results:**

The kinetics and the timings of B- and T-cell release from the bone marrow and thymus during antiretroviral therapy were substantially different, with a decreased B-cell release and an increased thymic output after the prolonged therapy. The multivariable regression analysis showed that a longer pre-therapy infection duration predicts a minor TREC increase and a major KREC reduction.

**Conclusions:**

The quantification of KRECs and TRECs represents an improved method to monitor the effects of therapies capable of influencing the immune cell pool composition in HIV-1-infected patients.

## Background

Although CD4^+^ T cells are the major target of HIV-1, this infection widely impairs the viability and function of numerous other immune cells [1]. In particular, in the absence of therapy, HIV-1 infection is associated with several B-cell defects, including polyclonal hypergammaglobulinemia [2], modified expression of activation and costimulatory markers [3-6], decreased B-cell survival [7,8], and the presence of exhausted terminally differentiated B cells or CD27^-^ memory B cells [9-11]. Furthermore, recent results showed that HIV-1 infection not only induces a strong depletion in memory B cells, but is also associated with defects in the naive B-cell subset [12].

Combined antiretroviral therapy (cART) is very efficient in reducing HIV-1 load and, currently, even with salvage therapy, up to 90% of treated HIV-1-infected adults attain viral RNA plasma levels under the limit of detection of commercially available tests [13]. As a consequence of the viral suppression, resulting into a gradual reprise of thymic output, the CD4^+^ cell count reaches normal levels in most but not all treated patients [14]. Still, in some of them, the T-cell recovery remains abnormally low in spite of the complete suppression of viral replication, and they are at increased risk of disease progression and death [15-17]. Therefore, one of the problems in the field of anti-viral therapy in HIV-1-infected patients is how to achieve an efficient monitoring of the immune reconstitution following cART. Routinely, the immune system restoration is evaluated by T-cell phenotyping. A more specific way to measure the recovery of the immune system is the quantification of the recent thymic emigrants (RTE) that are CD4^+^ lymphocytes expressing the CD45RA and CD31 markers or harbouring the T-cell receptor excision circles (TRECs), which are extrachromosomic circular DNA episomes produced during T-cell receptor rearrangement. TRECs, in particular, have been used as a surrogate marker of thymic output [18]. While TREC number in HIV-1-infected patients has been found to correlate with different clinical-pathological parameters (age, plasma HIV-1 RNA, CD4^+^ T-lymphocyte counts, CD4^+^ T-lymphocyte percentages, and naive CD4^+^ T-lymphocyte number) and TREC number of HIV-1-infected children increases during cART [19-22], to our knowledge, no studies have investigated the effects of cART treatment on the release of new B lymphocytes from the bone marrow of treated patients. Moreover, it is not known whether the recovery of B and T cells occurs simultaneously. Therefore, here, the effect of cART on the mobilization of new B and T cells during a long follow-up (72 months) was analyzed by a duplex real-time PCR that combines the measure of TRECs with the quantification of the “K deleting recombination excision circles” (KRECs) that assesses the extent of the B-cell output [23,24]. Real-time PCR was also used to quantify the mRNA expression of interleukin 7 (IL-7) and of the alpha chain of IL-7 receptor (IL-7Rα), while flow cytometry was used to evaluate the cell surface expression of IL-7Rα on CD4^+^ cells and the modulation of B- and T-cell subsets.

## Methods

### Participants and study design

Thirty-six HIV-1-infected adult patients (group I), enrolled by the Institute of Infectious and Tropical Diseases of University of Brescia (Italy) during the SImplified Sequencing THERapy trial (SI.S.THER.), participated to this study. SI.S.THER. was a 12 months long multicentre prospective randomized trial, in which HIV^+^ patients who had never been treated before, but requiring antiretroviral therapy according to the current guidelines for the use of antiretroviral therapy, were randomly assigned, in a one-to-one ratio, to receive either zidovudine + lamivudine + lopinavir/ritonavir or tenofovir + lamivudine + efavirenz [25]. Peripheral blood mononuclear cells (PBMCs), prepared by Ficoll-Hypaque density gradient centrifugation, were obtained from the blood of these patients before therapy initiation (T0), after 6 (T6) and 12 (T12) months, and then stored and used for laboratory analysis. A further blood sample was obtained after 6 years of antiretroviral therapy (T72). The routine clinical examination, CD4^+^ T-cell count, and HIV-1 RNA quantification were assessed at all time points. A first control group (group II) consisted of 22 randomly selected HIV-1-infected patients who were regularly followed up in the HIV outpatient clinic, but did not need antiretroviral therapy according to the current guidelines. HIV-uninfected persons, matched by age and gender with the patients of group I in a two-to-one ratio, were selected as a second control group (group III). Only one blood sample was obtained from individuals of group II and III. The baseline characteristics of patients of group I and II and the immuno-virological features observed during the follow-up in group I are summarized in Table 1. 

**Table 1:** **Baseline characteristics of HIV-1**^**+**^**patients**

	**Group I**				**Group II**	p -value*
	**T0**	**T6**	**T12**	**T72**		
Males	32 (88.9)	-	-	-	17 (77.3)	-
Age (years)	39 (35–45)	-	-	-	38 (33–43)	-
Risk factors for HIV-1:						
· Heterosexual	20 (55.6)	-	-	-	7 (31.8)	-
· Homo/bisexual	10 (27.8)	-	-	-	10 (45.5)	-
· IVDU	3 (8.3)	-	-	-	5 (22.7)	-
Other/unknown	3 (8.3)	-	-	-	0 (0)	-
CD4^+^ T cells (cells/μL)	258 (139–331)	377 (225–505)	419 (247–570)	688 (407–822)	538 (455–691)	<0.0001
CD8^+^ T cells (cells/μL)	754 (637–1338)	781 (598–990)	753 (561–983)	707 (598–805)	757 (595–1138)	-
CD4^+^/CD8^+^ T-cell ratio	0.3 (0.2–0.4)	0.5 (0.3–0.7)	0.6 (0.4–0.7)	0.8 (0.6–1.1)	0.7 (0.5–1)	<0.0001
HIV-1 RNA (copies/mL)	74533 (21264–196233)	< 50 (50–50)	< 50 (50–50)	< 37 (37–37)	14155 (263–33380)	<0.01

For continuous variables the medians (interquartile ranges) are shown, while for categorical variables the number (percentage) is shown.

Group I, HIV-1-infected patients enrolled in the SI.S.THER. study; group II, HIV-1-infected patients naive to antiretroviral therapy.

IVDU indicates Intravenous Drug Users.

*p -value of the comparison between group I at T0 and group II.

The study was conducted in accordance with good clinical practice (ICH-E6). The trial and amendments received approval by the institutional review board/independent ethics committee (resolution of n° 33 of March 11, 2011) and the patients provided written informed consent before the SI.S.THER. trial and for the present study as well.

### Real-time PCR for KRECs, TRECs, IL-7 and IL-7Rα quantification

DNA was extracted from about 3 × 10^6^ PBMCs using the QIAamp DNA Blood Mini Kit (Qiagen, Valencia, CA), according to the manufacturer’s instructions. KREC and TREC molecules were detected by a duplex quantitative real-time PCR, in which the simultaneous amplification of the two target sequences occurs in a single reaction, and the specific probes, labeled with different fluorescent dyes, allowed to separately quantify the amount of each target. The sequence of primers and probes for the signal joint regions of KRECs and TRECs, as well as for the reference gene, which was a fragment of the T-cell receptor alpha constant gene (TCRAC), have been previously described [23]. The PCR reactions were developed in 96-well optical reaction plates (Applied Biosystems, Foster City, CA) and the reaction mixture was prepared in a total volume of 25 μL containing 12.5 μL of 2x TaqMan Universal PCR master mix containing AmpErase UNG (Applied Biosystems), 900 nM forward and reverse primers, 200 nM probes, and 5 μL of genomic DNA solution. Amplification of the TCRAC was done in the same plate. The conditions for the real-time PCR, performed on a 7500 Fast Real-Time PCR System (Applied Biosystems), were 50°C for 2 minutes and 95°C for 10 minutes, followed by 45 cycles of 95°C for 15 seconds and 60°C for 1 minute. All samples were measured in duplicate. The number of KREC, TREC and TCRAC molecules in the sample was extrapolated by the standard curve obtained by serial dilutions (10^6^, 10^5^, 10^4^, 10^3^, 10^2^, and 10) of a linearized plasmid DNA, containing three inserts corresponding to fragments of KRECs, TRECs and TCRAC, which were amplified in each PCR plate. KREC and TREC copies were calculated per mL of blood by multiplying the number of KREC or TREC molecules in the sample by the lymphocyte plus monocyte count per mL of blood, as done by Chen et al. [26]. The quantity of TRECs per 10^6^ PBMC was also reported, and their density per naive cells or per RTE cells was calculated dividing the value of TRECs/mL by the numbers of naive T cells/mL or RTE cells/mL.

One hundred and fifty ng of total RNA, extracted from PBMCs using NucleoSpin RNA II kit (Macherey-Nagel GmbH & Co. KG, Düren, Germany) were reverse transcribed into cDNA using random hexamers and Taqman reverse transcription reagents (Applied Biosystems). An equivalent of 22.5 ng of RNA was analyzed in each well on the 7500 Fast Real-Time PCR System using TaqMan Gene Expression Assay for IL-7 and IL-7Rα, (IL-7: Hs00174202_m1 and IL-7R: Hs00902334_m1; Applied Biosystems) or primers and probe for glyceraldehyde 3-phosphate-dehydrogenase (GAPDH: forward primer: 5′GAAGGTGAAGGTCGGAGTC3′, reverse primer: 5′GAAGATGGTGATGGGATTTC3′ and probe: Fam-CAAGCTTCCCGTTCTCAGCC-Tamra) synthesized according to the Applied Biosystems recommendations. Results, obtained using the comparative Cycle threshold (Ct) method with GAPDH as the reference gene and the RNA extracted from the pooled PBMCs of 10 healthy donors as the calibrator sample, were reported as the value of “– ΔΔCt”, being ΔΔCt = [(Ct target – Ct reference gene) _patient_ – (Ct target – Ct reference gene) _calibrator_].

### Cytofluorimetric characterization of lymphocyte subpopulations and IL-7Rα analysis

B- and T-cell subsets were determined by six-colour flow cytometry analysis on the fresh whole blood obtained at T72 from group I patients and from both group II and III control individuals. Briefly, for the identification of B-cell subsets, 100 μL of whole blood was stained with various combinations of optimal staining concentrations (previously determined by titrations) of peridin-clorophyll protein-Cy5.5 anti-CD19, phycoerythtin-Cy7 anti-CD10, fluorescein isothiocyanate anti-IgD, and phycoerythrin anti-CD27 (BD Pharmingen, Heidelberg, Germany) monoclonal antibodies (mAbs). For T-cell subpopulation characterization the following mAbs were used: allophycocyanin-H7 anti-CD4, fluorescein isothiocyanate anti-CD45RA (BD Pharmingen), peridin-clorophyll protein-Cy5.5 anti-CCR7 (BioLegend, San Diego, CA), and allophycocyanin anti-CD31 (Miltenyi Biotec, Bergisch Gladbach, Germany) mAbs. The staining was performed for 15 minutes at room temperature in the dark. Then, red blood cells were lysed with BD Pharm Lyse™ lysing solution (BD Pharmingen). Data were collected immediately using a 6-colour 2-laser BD FACSCanto II cytometer and analyzed with the FACSDiva software (BD Biosciences, San Jose, CA). B- and T-cell subsets were identified (as previously reported [27-30] and shown in the Additional file 1: Figure S1) as: CD19^+^CD10^+^ immature, CD19^+^CD10^−^IgD^+^CD27^−^naive mature, CD19^+^CD10^−^IgD^−^CD27^+^ memory switched and CD19^+^CD10^−^IgD^+^CD27^+^ memory unswitched B cells; CD4^+^CD45RA^+^CCR7^+^ naive, CD4^+^CD45RA^+^CCR7^+^CD31^+^ RTE, CD4^+^CD45RA^−^CCR7^+^ central memory (T_CM_) and CD4^+^CD45RA^−^CCR7^−^ effector memory (T_EM_) T cells. The level of cell surface expression of IL-7Rα was studied by analyzing the median fluorescence intensity (MFI) of PBMCs incubated with anti-CD4 and phycoerythrin-Cy7 anti-CD127 (eBioscience, San Diego, CA) mAbs.

### Statistical analysis

Univariate comparisons between log KREC, log TREC, IL-7, and IL-7Rα means, calculated in group I, II and III (and reported in the manuscript along with their standard deviation), were performed by ANOVA followed by the Student-Newman-Keuls test for all the pairwise comparisons, whereas changes of their mean values over the follow-up were assessed by repeated measure ANOVA, followed by Bonferroni-corrected multiple tests. A mixed-model ANOVA for repeated measures was used to compare the between-group differences in TRECs, KRECs or CD4 during the follow-up time, and planned orthogonal contrasts (with Bonferroni-corrected p-values) were performed to compare the group means at the time points of interest. Spearman’s rank correlation coefficients were used to assess the correlations of the immunological parameters with age. To evaluate the impact of the immunological and virological parameters on therapy-induced KREC or TREC changes in patients of group I, two multivariable linear regression models were fitted, after performing univariable linear regressions aimed at identifying significant factors and at excluding confounding ones. The differences between the log-value of KRECs/mL and TRECs/mL found at T72 and T0 were chosen as the dependent variables. As independent variables were tested the basal levels of several immune parameters that could have affected the immune recovery, as listed in Table 2. As a result, the following covariates, whose p-value resulted at least < 0.10, were chosen as predictors in the final multivariable models: the pre-therapy level of log KRECs/mL or log TRECs/mL (which were included in order to adjust for their potential confounding value), the infection duration (estimated from the presumed time of infection, which corresponds to the date of the first positive HIV-1 test), the pre-therapy viremia, and the pre-therapy CD4^+^/CD8^+^ cell ratio. The CD4^+^ cell count was excluded because it was highly correlated to the CD4^+^/CD8^+^ cell ratio. Accordingly, the obtained multivariable regression coefficients represented the variation of log KREC or log TREC differences from T72 to T0, per unit change of the listed independent variables (Table 3); their values are reported in the manuscript, after anti-log transformation, as the percent change of log KREC or log TREC T72-to-T0 variations. The Kruskal-Wallis test, followed by the Dunn’s post-hoc test, was employed to compare flow cytometry results, which are reported herein as medians and depicted as box-and-whisker plots. Unless otherwise specified, the criterion for statistical significance was set at p < 0.05.

**Table 2:** Univariate regression modelling KRECs and TRECs change from T0 to T72

	**T72-T0 log KRECs/mL difference**	**T72-T0 log TRECs/mL difference**
	**coefficient**	**coefficient**
log KRECs/mL at T0	−0.39 [−0.795,0.0155]*	n.i.
log TRECs/mL at T0	n.i.	−0.67 [−0.826,−0.514]‡
Age at T0	0.000488 [−0.0297,0.0307]	0.00908 [−0.0367,0.0549]
Viremia at T0 (log DNAcopies/mL)	0.07 [−0.136,0.276]	−0.0774 [−0.378,0.223]
CD4 at T0 (cells/mL)	0.000225 [−0.00117,0.00162]	−0.00171 [−0.00363,0.000212]*
CD4/CD8 at T0	0.548 [−0.451,1.55]	−1.88 [−3.18,−0.585]†
Therapy arm	0.156 [−0.196,0.507]	−0.0792 [−0.594,0.435]
Infection duration before therapy start (years)	−0.105 [−0.163,−0.0471]†	−0.0753 [−0.172,0.0217]
HCV coinfection	−0.131 [−0.634,0.372]	−0.470 [−1.141,0.200]

95% confidence intervals are in square brackets; n.i.: not included in the model; * p <0.10; † p <0.01; ‡ p <0.001.

**Table 3:** Multivariable regression coefficients of covariates predicting KREC and TREC changes from T0 to T72

	**T72-T0 log KRECs/mL difference**	**T72-T0 log TRECs/mL difference**
	**coefficient**	**coefficient**
log KRECs/mL at T0	−0.473 [−0.775,−0.172]†	n.i.
log TRECs/mL at T0	n.i.	−0.558 [−0.724,−0.391]‡
Viremia at T0 (log DNAcopies/mL)	n.i.	−0.211 [−0.373,−0.0482]†
CD4/CD8	0.868 [0.155,1.58]†	−0.769 [−1.6,0.057] *
Infection duration before therapy start (years)	−0.111 [−0.16,−0.0629]‡	−0.0648 [−0.118,−0.0121]†
R-squared	0.568	0.820

95% confidence intervals are in square brackets; n.i.: not included in the model. * p <0.10; † p <0.01; ‡ p <0.001.

## Results

### Quantification of KRECs and TRECs

**Figure 1:**
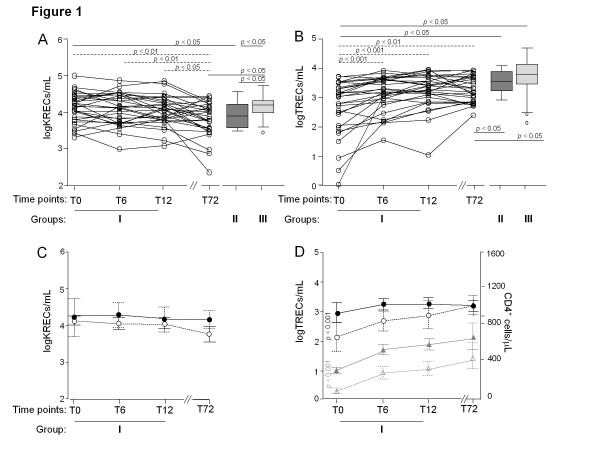
**Quantification of KRECs and TRECs.** (**A**) Number of KREC^+^ lymphocytes in 36 HIV-1^+^ patients that initiated cART and were thereafter followed up for 72 months (group I, from T0 to T72), in 22 HIV-1^+^ patients that did not need therapy (group II), and in 72 age and gender-matched uninfected subjects (group III). (**B**) Number of TREC^+^ lymphocytes in group I, II and III. Values in individual patients are shown as clear circles, and dashed significance lines indicate the results of the longitudinal analysis. (**C**) KRECs and (**D**) TRECs and CD4^+^ cell modulation during the follow-up in HIV-1^+^ patients of group I divided according to their CD4^+^ cell number before therapy start (< 200/μL: clear symbols, dashed lines; and ≥ 200/μL: filled symbols, solid lines). The lines (in gray for CD4^+^ cells) connect the means (indicated either by circles, as in the case of KRECs and TRECs, or by triangles, as in the case of CD4^+^ cells); error bars indicate the 95% confidence intervals.

The number of KRECs and TRECs of HIV-1-infected patients (group I: subjects treated with cART and followed up for 72 months; group II: HIV-1-infected patients naive for therapy) was compared to that of matched HIV-1 uninfected controls (group III). KRECs of patients of group I at T0 (4.15 ± 0.37 log KRECs/mL) were similar to those of group III (4.13 ± 0.27 log KRECs/mL) and significantly higher than those of group II (3.94 ± 0.35 log KRECs/mL, p < 0.05; Figure 1A). In addition, the number of KRECs found at T0 was not modified by 6 or 12 months of therapy (T6: 4.09 ± 0.42, T12: 4.07 ± 0.39 log KRECs/mL, p = NS), while the long-lasting treatment resulted in a significant decrease in new B-cell release from the bone marrow (T72: 3.84 ± 0.49 log KRECs/mL, p *<* 0.01), so that KREC^+^ cells of group I at T72 became significantly lower than those of group III (p < 0.05), but remained similar to those of group II (p = NS). The number of KRECs was not correlated with increasing age in any of the groups (data not shown), while only in group I it was positively correlated with the number of CD4^+^ cells before starting cART (T0; r = 0.38; p < 0.05) and after 6 months of therapy (T6; r = 0.51; p < 0.01), but not after one year of treatment (T12) or at the end of the follow-up (T72).

The number of TREC^+^ cells, before starting cART, was significantly lower in group I than in both control group II and III (2.67 ± 0.87 log TRECs/mL vs. 3.53 ± 0.37 log TRECs/mL, p < 0.05 and vs. 3.71 ± 0.55 log TRECs/mL, p < 0.05; Figure 1B), and no correlation between TRECs and patient age was observed in this group (data not shown). In contrast, and also differently from KRECs, TRECs decreased with older age both in group II (r = −0.62; p < 0.001) and in group III (r = −0.46; p < 0.001), with an average TREC^+^ cell loss of 55% every 10 years. Moreover, the number of TREC-containing cells of group I, but not of group II, was positively correlated to the number of CD4^+^ cells at T0 (r = 0.57 p < 0.01), T6 (r = 0.40; p < 0.01) and T12 (r = 0.41; p < 0.01), but not at T72. An increase of TREC^+^ cell production in group I was already evident at 6 months of therapy (T0: 2.67 ± 0.87 log TRECs/mL vs. T6: 3.09 ± 0.59 log TRECs/mL, p < 0.001) and persisted also after the long-lasting treatment (T72: 3.22 ± 0.44 log TRECs/mL, p < 0.01), but it never reached the levels observed in control group II or III (3.53 ± 0.37 log TRECs/mL and 3.71 ± 0.55 log TRECs/mL, respectively; p < 0.05). A similar pattern of TREC modulation was observed if TRECs were calculated per 10^6^ PBMC (see Additional file 1: Figure S2A); furthermore, the fold change increases from T0 to T6, T12 and T72 were comparable when TRECs were expressed in either forms (see Additional file 1: Figure S2B). Because patients of group I had a heterogeneous number of CD4^+^ cells that, at T0, was correlated to KREC and TREC levels, they were stratified into two groups: patients with a CD4^+^ cell count lower (21 out 36) or higher (15 out 36) than 200 cells/μL. While KRECs did not show a different trend in the two groups at any time point (Figure 1C), the level of TREC was differentially modulated, with a pattern not reflecting that of CD4^+^ cell increase. Indeed, at T0, TRECs of patients with CD4^+^ cells < 200/μL were the lower (2.21 log TRECs/mL vs. 2.99 log TRECs/mL, p < 0.001), but at T72 they increased up to the level found in patients with CD4^+^ cells > 200/μL (3.20 log TRECs/mL vs 3.22 log TRECs/mL, p = NS), whose TRECs, on the other hand, had remained substantially unchanged (Figure 1D). In contrast, the total CD4^+^ cell count grew very similarly in the two groups, thus remaining significantly different at all time points (p < 0.001; Figure 1D).

### Measure of IL-7 and IL-7Rα

**Figure 2:**
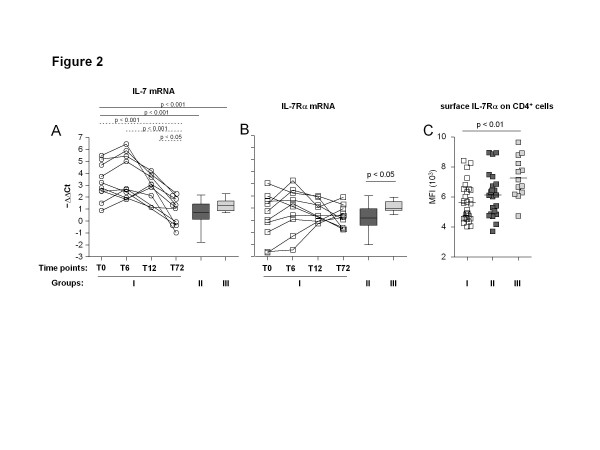
**Quantification of IL-7 and IL-7Rα mRNA and IL-7Rα cell surface expression.** (**A**) Quantification of IL-7 mRNA in PBMCs of 10 HIV-1^+^ patients that initiated cART and were thereafter followed up for 72 months (group I, from T0 to T72), of 18 HIV-1^+^ patients that did not require therapy (group II), and of 11 uninfected subjects (group III). Values measured in individual patients are shown as clear circles. Dashed significance lines indicate the results of the longitudinal analysis within patients of group I. (**B**) RNA expression of IL-7Rα in PBMCs of patients of groups I, II and III. Values measured in individual patients are shown as clear squares. (**C**) Surface expression of IL-7Rα on CD4^+^ cells in the three groups of subjects as determined by flow cytometry; statistical analysis performed by ANOVA followed by the Bonferroni test.

To evaluate whether IL-7 and IL-7Rα could be involved in the modulation of KRECs and TRECs of cART treated patients, we quantified the RNA of the two targets by real-time PCR in 10 patients of group I. These cART-treated patients showed a wide range of IL-7 RNA values: the mean level of IL-7 RNA relative expression, which, before therapy, was significantly higher than that of subjects of group II and III (T0: 3.26 ± 1.53 vs. group II: 0.69 ± 0.96, p < 0.001; and vs. group III: 1.32 ± 0.52, p < 0.001), further increased after six months, but then decreased to reach the levels observed in both groups II and III after 6 years of therapy (T72: 0.81 ± 1.17, p < 0.001; Figure 2A). The levels of RNA for IL-7Rα in group I were also highly heterogeneous at the first two time points of the follow-up, then the range of values tended to narrow at the following time points, but their mean value did not significantly change over time, nor there were any significant differences in comparison to that of patients of group II and controls of group III at any time point. However, the mean IL-7Rα RNA level was lower in patients of group II than in group III (0.16 ± 1.03; 1.16 ± 0.47, p < 0.05; Figure 2B). Furthermore, the level of IL-7Rα cell surface expression, calculated as the MFI of anti-CD127 mAb on CD4^+^ T cells, was lower in the patients treated for 6 years with cART than in patients of group II and subjects of group III (5630 ± 1250 vs. 6157 ± 1498 vs. 7300 ± 1375 MFI; Figure 2C), even though the difference was significant only in comparison to group III (p < 0.01). These data are in agreement with those indicating that HIV-1 infection is associated with decreased IL-7Rα expression on circulating T cells, and effective antiretroviral therapy only partially restores this defect [31,32]. 

### Identification of factors influencing KREC and TREC production

To better assess how the immunological or virological parameters affected the observed therapy-induced KREC or TREC changes, we employed the linear regression analysis. First, a series of univariable regressions were fitted in order to identify significant covariates and putative confounding variables. Results showed that a lower increase of log TRECs/mL from T0 to T72 is to be expected in the presence of a higher basal log TRECs/mL or of a higher CD4^+^/CD8^+^ ratio at T0, and that greater log KRECs reductions could be ascribed to longer infection duration before therapy initiation. Of note, neither the different therapy arm nor the presence of HCV co-infection affected KREC and TREC changes (Table 2).

The effects of these variables were then reciprocally adjusted by fitting multivariable regressions (Table 3), which, after anti-log conversion of the obtained coefficients, indicated that the TREC increase over time was 72% lower for each additional 1-log of basal TREC value and 38% lower for each 10-fold increase of blood HIV-1-RNA. At the same time, a higher basal level of log KRECs/mL accounted for a stronger reduction in the release of new B cells from the bone marrow following the cART, in the amount of a 66% decrease for each additional 1-log of basal KRECs/mL, whereas higher basal CD4^+^/CD8^+^ ratios determined a tapering of KREC decrease in the measure of 22% for each 0.10-higher CD4^+^/CD8^+^ ratio. Furthermore, for any additional year of infection duration at the moment of therapy start, the decrease of KRECs from T0 to T72 was enhanced by 23%, whereas the increase of TRECs was reduced by a further 14%.

### Cytofluorimetric analysis of B- and T-cell subsets

**Figure 3:**
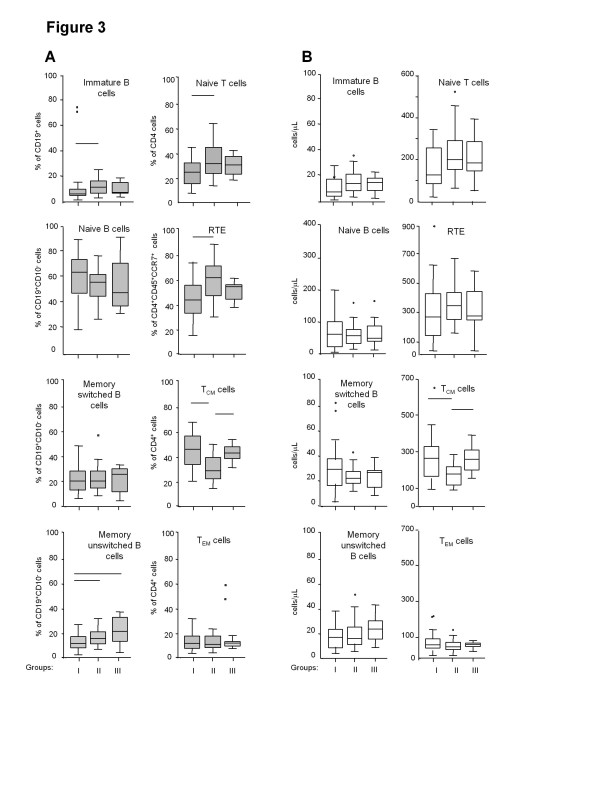
**Quantification of B- and T-cell subpopulations.** (**A**) Percentage (gray boxes) and (**B**) number (clear boxes) of immature, naive, memory switched, and memory unswitched B cells, as well as of naive, recent T emigrants (RTE), central memory (T_CM_) and effector memory (T_EM_) T cells in patients treated for 72 months with cART (group I), patients not requiring therapy (group II), and non-infected individuals (group III). Horizontal lines indicate the significant differences (all p < 0.05).

The percentage (Figure 3A) and number (Figure 3B) of B- and T-cell subsets were determined by cytofluorimetric analysis on fresh blood samples of the HIV-1-infected patients of group I obtained after 72 months of therapy, which were compared to those of subjects of group II and group III. Patients of group I had a lower percentage of immature B cells with respect to those of group II (6.0% vs. 12.1%, p < 0.05), a percentage and number of naive and memory switched B cells similar to those of the other two groups (p = NS) and a lower percentage of memory unswitched B cells in comparison to that of individuals of group II and III (12.54% vs. 16.2% vs. 22.0%, respectively, p < 0.05). Within the T-cell compartment, the percentage, but not the number, of naive cells and of RTE, which are the cells that were recently released from the thymus, was lower in group I than in group II (23.4% vs. 33.4%; p < 0.05), but similar to that of group III (29.0%; p = NS). Since changes in proliferation or loss rate occurring within naive and RTE cells may interfere with the measure of thymic export, the number of TRECs within naive and RTE populations of untreated patients of group I and II was evaluated. As shown in the Additional file 1: Figure S3A, TRECs were not different (p = NS) in the two T-cell subsets. Furthermore, the slope and intercept of the regression lines, obtained by fitting a model with TRECs/mL as dependent variable and naive/mL or RTE/mL as independent variables, were similar (see Additional file 1: Figure S3B). Therefore, it is likely that no significant variations occurred in the number of naive and RTE cells that would induce a misinterpretation of TREC data.

Patients of group I showed a significantly higher percentage and number of T_CM_ lymphocytes in comparison to those of group II subjects (43.5% vs. 27.1%, p < 0.05; and 265 cells/μL vs. 175 cells/μL, p < 0.05; Figure 3A, B), who, in turn, had a significantly lower percentage and number of these cells in respect to the healthy individuals of group III (27.1% vs. 40.5%, p < 0.05; and 175 cells/μL vs. 255 cells/μL, p < 0.05). No significant differences were observed for T_EM_ cells in the three groups.

## Discussion

The extent of thymic output is one of the most important markers of the T-cell immune recovery in HIV infection, but this information remains incomplete if not complemented with the assessment of B-cell compartment that is known to be also impaired during HIV infection. Indeed, in HIV-1-infected individuals, the loss of memory B cells, together with an altered differentiation of naive B cells, results in the production of a low quantity of antigen specific antibodies [33]. However, given the various B-cell sources, routine serotyping does not allow discrimination between antibodies produced by newly developed B cells and those produced by old mature B cells that have been expanded in the periphery. If the antibody production is solely based on expanding mature B cells, antibody production will end as the old B cells die off. Therefore, the integrity and efficacy of regeneration of the B-cell compartment in HIV^+^ patients should also be monitored by distinguishing newly produced B cells from the old ones. We had previously validated a method based on the quantification of KRECs and TRECs which appears to provide a reliable quantification of both newly produced B and T cells [23,24]. Thus, we applied the KREC/TREC assay to monitor the immunoreconstitution of HIV-1^+^ patients. Following what suggested by Ribeiro and Perelson [34] and Lorenzi et al. [35], we expressed the results of our analysis in terms of KRECs/TRECs per mL of blood in order to overcome the bias related to the extensive peripheral cell divisions that would dilute TREC content and make any KREC/TREC values calculated “per 10^6^ unsorted PBMCs” difficult to interpret, in particular in subjects with a chronic immune activation and increased peripheral lymphocyte proliferations, such as HIV-infected patients.

While patients of group I at T0 and of group II were all not undergoing cART, different levels of KRECs and TRECs were found in the two groups. However, these patients represent two different types of naive subjects because those of group II still maintained a quite conserved immune system (CD4^+^ cell count >350/μL) and did not need antiretroviral therapy according to the current guidelines for the diagnosis and treatment of HIV-1 infection, whereas those of group I at T0 were patients with a number of CD4^+^ T cells <350/μL or with clinical AIDS-defining conditions at diagnosis. They were diagnosed with HIV-1 infection when the disease had already progressed to a severe level of immune deterioration, so that after starting antiretroviral therapy and despite full viral suppression, some of them showed a suboptimal immune recovery.

We also found that the kinetics and the timings of B- and T-cell release from the bone marrow and thymus during cART are completely different. KREC production, that remains unchanged until one year of therapy, decreases following prolonged therapy to levels that were similar to those of therapy-free HIV-1^+^ patients, but lower than those of uninfected subjects. This result is not unexpected since it has been previously demonstrated that, in patients with idiopathic CD4^+^ lymphocytopenia, the percentage of immature/transitional B cells, that are known to contain the highest number of KRECs [36], is inversely correlated with the CD4^+^ T-cell count [37]. Accordingly, in our patients, the lowest levels of KRECs were observed in the long-lasting treated patients who also showed the higher CD4^+^ lymphocyte count. Furthermore, in patients with idiopathic CD4^+^ lymphocytopenia, the expansion of immature/transitional B cells appears to be associated with elevated serum levels of IL-7 [37]. Previous studies have shown a significant increase of IL-7 level also in lymphopenic states due to HIV-1 infection and currently there are ongoing clinical investigations on IL-7 as a potential anti-HIV therapy [38,39]. In HIV-infected patients, the administration of IL-7 increases in the proportion of immature/transitional B cells [39]. Thus, the low release of B cells from the bone marrow we have observed in our patients after prolonged cART may be related to the levels of IL-7 that, although being very high in the patients that initiated cART, progressively decreased to levels comparable to those observed in patients not requiring therapy and healthy controls. Therefore, since we also found that prolonged cART induces an increase of thymic output, the concomitant administration of cART and IL-7 may represent a novel strategy for improving the immune reconstitution in chronic HIV infection. In this regard, KREC and TREC quantification could be a useful tool for monitoring the efficacy and safety of IL-7-based therapies [40]. In addition, the observed decrease of memory unswitched B cells (feature that completes data by Chong et al. [41], reporting a decline in total memory B cells), together with the decline in new B-cell production, is likely to contribute to the reduced humoral immune response observed in HIV-1^+^ patients despite antiretroviral treatment.

As already reported by several authors [42,43], we found that TREC production was increased following cART initiation, reached a plateau after 12 months, and remained stable during several years of therapy, although never reaching the levels observed in the HIV-1-infected patients not requiring therapy or in the uninfected controls. We also observed the known dependency of TRECs on ageing [35] only in HIV-uninfected controls and in the HIV-1-infected patients that did not need therapy, but not in those who underwent cART. This is likely to be due to an incomplete immune reconstitution as indicated by the persistent low number of CD4^+^ cells found in several patients of this group. Furthermore, the increase in TRECs appears to be more evident in patients with a lower viral load, a lower CD4^+^/CD8^+^ ratio, or lower CD4^+^ cell counts before therapy beginning. In particular, before cART, TRECs were significantly lower in those patients with CD4^+^ cells < 200/μL than in those with CD4^+^ cells > 200/μL, although they increased only in the patients with low CD4^+^ cells, so that the difference was abolished after prolonged therapy. The two anti-viral drug combinations appear not to affect the extent of new B- and T-cell mobilization that, on the contrary, was affected by the duration of the infection before therapy initiation. Indeed, each year of infection before therapy weakens the recovery of TRECs and favors the decrease of KRECs observed in most patients after treatment. While it has been previously reported that the early initiation of an antiretroviral therapy restores the memory B-cell number, though only temporarily [44,45], to our knowledge this is the first report linking the extent of B- and T-cell release from the production site following long-lasting antiretroviral therapy to the duration of HIV-1 infection before treatment initiation.

Flow cytometry results partially overlap those obtained by real-time PCR, because they confirm the lower levels of immature B cells, which are, for the most part, recently produced B cells, and of naive and RTE T lymphocytes in long-lasting treated patients in respect to patients that did not need therapy (but only if calculated as percentage and not as total number). Furthermore, the increase of T_CM_ cells already observed by Hodge et al. [46] after 2 years of antiretroviral therapy, seems to persist also after long-lasting treatment.

## Conclusions

In conclusion, the analysis of KRECs and TRECs appears to be a valuable aid for the fine immunological characterization of HIV-1^+^ patients treated with cART. Furthermore, because the administration of recombinant IL-7 in humans increases the number of TREC-containing cells [47] and high IL-7 serum levels appear to be associated with the expansion of immature/transitional B cells, our combined TREC and KREC assay could be useful for the monitoring of IL-7 based therapies.

## Abbreviations

cART : Combined antiretroviral therapy; KRECs : K-deleting recombination excision circles; IL-7 : Interleukin 7; IL-7Rα : Interleukin 7 receptor alpha; PBMCs : Peripheral blood mononuclear cells; RTE:Recent thymic emigrants; SI.S.THER.:SImplified Sequencing THERapy trial; T_CM_: Central memory T cells; T_EM_: Effector memory T cells; TCRAC : T-cell receptor alpha constant gene; TRECs : T-cell receptor excision circles.

## Competing interests

The authors declare that they have no competing interests.

## Authors’ contributions

EQ participated at the design and conceptualization of the study, recruited patients, and revised the manuscript. FS performed the statistical analysis and drafted the manuscript. MC performed the flow cytometric analysis. CZ and AS performed the molecular biology experiments. DG assisted in identifying patients, collected the data, and revised the manuscript. CT recruited patients and revised the manuscript. LC obtained the funding and revised the manuscript. LI obtained the funding, supervised the study, analyzed data, and wrote the manuscript. All authors read and approved the final manuscript.

## Supplementary Material

Additional file 1 Contains Figure S1, S2, and S3 (legend and artwork). Click here for file

## References

[B1] Belyakov IM Berzofsky JA Immunobiology of mucosal HIV infection and the basis for development of a new generation of mucosal AIDS vaccines Immunity 2004 20 247 253 10.1016/S1074-7613(04)00053-6 15030769

[B2] Lane HC Masur H Edgar LC Whalen G Rook AH Fauci AS Abnormalities of B-cell activation and immunoregulation in patients with the acquired immunodeficiency syndrome N Engl J Med 1983 309 453 458 10.1056/NEJM198308253090803 6224088

[B3] Malaspina A Moir S Kottilil S Hallahan CW Ehler LA Liu S Planta MA Chun TW Fauci AS Deleterious effect of HIV-1 plasma viremia on B cell costimulatory function J Immunol 2003 170 5965 5972 12794123 10.4049/jimmunol.170.12.5965

[B4] Martínez-Maza O Crabb E Mitsuyasu RT Fahey JL Giorgi JV Infection with the human immunodeficiency virus (HIV) is associated with an in vivo increase in B lymphocyte activation and immaturity J Immunol 1987 138 3720 3724 2953790

[B5] Amadori A Chieco-Bianchi L B-cell activation and HIV-1 infection: deeds and misdeeds Immunol Today 1990 11 374 379 2103733 10.1016/0167-5699(90)90144-x

[B6] Moir S Ogwaro KM Malaspina A Vasquez J Donoghue ET Hallahan CW Liu S Ehler LA Planta MA Kottilil S Chun TW Fauci AS Perturbations in B cell responsiveness to CD4+ T cell help in HIV-infected individuals Proc Natl Acad Sci USA 2003 100 6057 6062 10.1073/pnas.0730819100 12730375 PMC156325

[B7] Samuelsson A Sönnerborg A Heuts N Cöster J Chiodi F Progressive B cell apoptosis and expression of Fas ligand during human immunodeficiency virus type 1 infection AIDS Res Hum Retroviruses 1997 13 1031 1038 10.1089/aid.1997.13.1031 9264290

[B8] De Milito A Mörch C Sönnerborg A Chiodi F Loss of memory (CD27) B lymphocytes in HIV-1 infection AIDS 2001 15 957 964 10.1097/00002030-200105250-00003 11399977

[B9] Ho J Moir S Malaspina A Howell ML Wang W DiPoto AC O’Shea MA Roby GA Kwan R Mican JM Chun TW Fauci AS Two overrepresented B cell populations in HIV-infected individuals undergo apoptosis by different mechanisms Proc Natl Acad Sci USA 2006 103 19436 19441 10.1073/pnas.0609515103 17158796 PMC1748244

[B10] Moir S Ho J Malaspina A Wang W DiPoto AC O’Shea MA Roby G Kottilil S Arthos J Proschan MA Chun TW Fauci AS Evidence for HIV-associated B cell exhaustion in a dysfunctional memory B cell compartment in HIV-infected viremic individuals J Exp Med 2008 205 1797 1805 10.1084/jem.20072683 18625747 PMC2525604

[B11] Nagase H Agematsu K Kitano K Takamoto M Okubo Y Komiyama A Sugane K Mechanism of hypergammaglobulinemia by HIV infection: circulating memory B-cell reduction with plasmacytosis Clin Immunol 2001 100 250 259 10.1006/clim.2001.5054 11465955

[B12] Richard Y Amiel C Jeantils V Mestivier D Portier A Dhello G Feuillard J Creidy R Nicolas JC Raphael M Changes in blood B cell phenotypes and Epstein-Barr virus load in chronically human immunodeficiency virus–infected patients before and after antiretroviral therapy J Infect Dis 2010 202 1424 1434 10.1086/656479 20874514

[B13] Yazdanpanah Y Fagard C Descamps D Taburet AM Colin C Roquebert B Katlama C Pialoux G Jacomet C Piketty C Bollens D Molina JM Chêne G ANRS 139 TRIO Trial Group High rate of virologic suppression with raltegravir plus etravirine and darunavir/ritonavir among treatment-experienced patients infected with multidrug-resistant HIV: results of the ANRS 139 TRIO trial Clin Infect Dis 2009 49 1441 1449 10.1086/630210 19814627

[B14] Vrisekoop N van Gent R de Boer AB Otto SA Borleffs JC Steingrover R Prins JM Kuijpers TW Wolfs TF Geelen SP Vulto I Lansdorp P Tesselaar K Borghans JA Miedema F Restoration of the CD4 T cell compartment after long-term highly active antiretroviral therapy without phenotypical signs of accelerated immunological aging J Immunol 2008 181 1573 1581 18606713 10.4049/jimmunol.181.2.1573

[B15] Tan R Westfall AO Willig JH Mugavero MJ Saag MS Kaslow RA Kempf MC Clinical outcome of HIV-infected antiretroviral-naive patients with discordant immunologic and virologic responses to highly active antiretroviral therapy J Acquir Immune Defic Syndr 2008 47 553 558 10.1097/QAI.0b013e31816856c5 18285713

[B16] Gutiérrez F Padilla S Masiá M Iribarren JA Moreno S Viciana P Hernández-Quero J Alemán R Vidal F Salavert M Blanco JR Leal M Dronda F del Amo J CoRIS-MD Patients’ characteristics and clinical implications of suboptimal CD4 T-cell gains after 1 year of successful antiretroviral therapy Curr HIV Res 2008 6 100 107 10.2174/157016208783885038 18336257

[B17] Antiretroviral Therapy Cohort Collaboration Life expectancy of individuals on combination antiretroviral therapy in high-income countries: a collaborative analysis of 14 cohort studies Lancet 2008 372 293 299 18657708 10.1016/S0140-6736(08)61113-7 PMC3130543

[B18] Douek DC McFarland RD Keiser PH Gage EA Massey JM Haynes BF Polis MA Haase AT Feinberg MB Sullivan JL Jamieson BD Zack JA Picker LJ Koup RA Changes in thymic function with age and during the treatment of HIV infection Nature 1998 396 690 695 10.1038/25374 9872319

[B19] Nobile M Correa R Borghans JA D’Agostino C Schneider P De Boer RJ Pantaleo G Swiss HIV Cohort Study De novo T-cell generation in patients at different ages and stages of HIV-1 disease Blood 2004 104 470 477 10.1182/blood-2003-12-4265 15059846

[B20] Ometto L De Forni D Patiri F Trouplin V Mammano F Giacomet V Giaquinto C Douek D Koup R De Rossi A Immune reconstitution in HIV-1-infected children on antiretroviral therapy: role of thymic output and viral fitness AIDS 2002 16 839 849 10.1097/00002030-200204120-00003 11919485

[B21] De Rossi A Walker AS Klein N De Forni D King D Gibb DM Increased thymic output after initiation of antiretroviral therapy in human immunodeficiency virus type 1-infected children in the Paediatric European Network for Treatment of AIDS (PENTA) 5 Trial J Infect Dis 2002 186 312 320 10.1086/341657 12134227

[B22] Chavan S Bennuri B Kharbanda M Chandrasekaran A Bakshi S Pahwa S Evaluation of T cell receptor gene rearrangement excision circles after antiretroviral therapy in children infected with human immunodeficiency virus J Infect Dis 2001 183 1445 1454 10.1086/320197 11329124

[B23] Sottini A Ghidini C Zanotti C Chiarini M Caimi L Lanfranchi A Moratto D Porta F Imberti L Simultaneous quantification of recent thymic T-cell and bone marrow B-cell emigrants in patients with primary immunodeficiency undergone to stem cell transplantation Clin Immunol 2010 136 217 227 10.1016/j.clim.2010.04.005 20452829

[B24] Serana F Sottini A Chiarini M Zanotti C Ghidini C Lanfranchi A Notarangelo LD Caimi L Imberti L The different extent of B and T cell immune reconstitution after hematopoietic stem cell transplantation and enzyme replacement therapies in SCID patients with adenosine deaminase deficiency J Immunol 2010 185 7713 7722 10.4049/jimmunol.1001770 21057082

[B25] Torti C Quiros-Roldan ME Cologni G Nichelatti M Ceresoli F Pinti M Nasi M Cossarizza A Lapadula G Costarelli S Manca N Gargiulo F Magoni M Carosi G Plasma HIV load and proviral DNA decreases after two standard antiretroviral regimens in HIV-positive patients naïve to antiretrovirals Curr HIV Res 2008 6 43 48 10.2174/157016208783571982 18288974

[B26] Chen X Barfield R Benaim E Leung W Knowles J Lawrence D Otto M Shurtleff SA Neale GA Behm FG Turner V Handgretinger R Prediction of T-cell reconstitution by assessment of T-cell receptor excision circle before allogeneic hematopoietic stem cell transplantation in pediatric patients Blood 2005 105 886 893 10.1182/blood-2004-04-1405 15358630

[B27] Sallusto F Lenig D Förster R Lipp M Lanzavecchia A Two subsets of memory T lymphocytes with distinct homing potentials and effector functions Nature 1999 401 708 712 10.1038/44385 10537110

[B28] Sims GP Ettinger R Shirota Y Yarboro CH Illei GG Lipsky PE Identification and characterization of circulating human transitional B cells Blood 2005 105 4390 4398 10.1182/blood-2004-11-4284 15701725 PMC1895038

[B29] Kimmig S Przybylski GK Schmidt CA Laurisch K Möwes B Radbruch A Thiel A Two subsets of naive T helper cells with distinct T cell receptor excision circle content in human adult peripheral blood J Exp Med 2002 195 789 794 10.1084/jem.20011756 11901204 PMC2193736

[B30] Chiarini M Sottini A Ghidini C Zanotti C Serana F Rottoli M Zaffaroni M Bergamaschi R Cordioli C Capra R Imberti L Renewal of the T-cell compartment in multiple sclerosis patients treated with glatiramer acetate Mult Scler 2010 16 218 227 10.1177/1352458509355460 20007428

[B31] Colle JH Moreau JL Fontanet A Lambotte O Joussemet M Delfraissy JF Theze J CD127 expression and regulation are altered in the memory CD8 T cells of HIV-infected patients–reversal by highly active anti-retroviral therapy (HAART) Clin Exp Immunol 2006 143 398 403 10.1111/j.1365-2249.2006.03022.x 16487237 PMC1809599

[B32] Koesters SA Alimonti JB Wachihi C Matu L Anzala O Kimani J Embree JE Plummer FA Fowke KR IL-7Ralpha expression on CD4+ T lymphocytes decreases with HIV disease progression and inversely correlates with immune activation Eur J Immunol 2006 36 336 344 10.1002/eji.200535111 16421946

[B33] De Milito A Nilsson A Titanji K Thorstensson R Reizenstein E Narita M Grutzmeier S Sönnerborg A Chiodi F Mechanisms of hypergammaglobulinemia and impaired antigen-specific humoral immunity in HIV-1 infection Blood 2004 103 2180 2186 10.1182/blood-2003-07-2375 14604962

[B34] Ribeiro RM Perelson AS Determining thymic output quantitatively: using models to interpret experimental T-cell receptor excision circle (TREC) data Immunol Rev 2007 216 21 34 17367332 10.1111/j.1600-065X.2006.00493.x

[B35] Lorenzi AR Patterson AM Pratt A Jefferson M Chapman CE Ponchel F Isaacs JD Determination of thymic function directly from peripheral blood: a validated modification to an established method J Immunol Methods 2008 339 185 194 10.1016/j.jim.2008.09.013 18854192 PMC2593795

[B36] van Zelm MC Szczepanski T van der Burg M van Dongen JJ Replication history of B lymphocytes reveals homeostatic proliferation and extensive antigen-induced B cell expansion J Exp Med 2007 204 645 655 10.1084/jem.20060964 17312005 PMC2137914

[B37] Malaspina A Moir S Chaitt DG Rehm CA Kottilil S Falloon J Fauci AS Idiopathic CD4+ T lymphocytopenia is associated with increases in immature/transitional B cells and serum levels of IL-7 Blood 2007 109 2086 2088 10.1182/blood-2006-06-031385 17053062 PMC1801046

[B38] Napolitano LA Grant RM Deeks SG Schmidt D De Rosa SC Herzenberg LA Herndier BG Andersson J McCune JM Increased production of IL-7 accompanies HIV-1-mediated T-cell depletion: implications for T-cell homeostasis Nat Med 2001 7 73 79 10.1038/83381 11135619

[B39] Sereti I Dunham RM Spritzler J Aga E Proschan MA Medvik K Battaglia CA Landay AL Pahwa S Fischl MA Asmuth DM Tenorio AR Altman JD Fox L Moir S Malaspina A Morre M Buffet R Silvestri G Lederman MM ACTG 5214 Study Team IL-7 administration drives T cell-cycle entry and expansion in HIV-1 infection Blood 2009 113 6304 6314 10.1182/blood-2008-10-186601 19380868 PMC2710926

[B40] Levy Y Sereti I Tambussi G Routy J Delfraissy J Molina J Fischl MA Croughs T Sekaly R Lederman M INSPIRE Study: Effects of r-hIL-7 on T cell recovery and thymic output in HIV-infected patients receiving c-ART - interim analysis of a phase I/IIa multicenter study. 49th Interscience Conference on Antimicrobial Agents and Chemotherapy, San Francisco, abstract H-1230a, 2009 [ http://www.abstractsonline.com/Plan/ViewAbstract.aspx?sKey=30cfb0e3-06f8-42ba-b4fb-47507d251418&cKey=c3c8cd19-6ca1-4b80-afe1-b802abba13b8&mKey={14EBFE7E-6F65-4D97-8CB6-F64F4347C38A}]

[B41] Chong Y Ikematsu H Kikuchi K Yamamoto M Murata M Nishimura M Nabeshima S Kashiwagi S Hayashi J Selective CD27+ (memory) B cell reduction and characteristic B cell alteration in drug-naive and HAART-treated HIV type 1-infected patients AIDS Res Hum Retroviruses 2004 20 219 226 10.1089/088922204773004941 15018710

[B42] Benveniste O Flahault A Rollot F Elbim C Estaquier J Pédron B Duval X Dereuddre-Bosquet N Clayette P Sterkers G Simon A Ameisen JC Leport C Mechanisms involved in the low-level regeneration of CD4+ cells in HIV-1-infected patients receiving highly active antiretroviral therapy who have prolonged undetectable plasma viral loads J Infect Dis 2005 191 1670 1679 10.1086/429670 15838794

[B43] Torti C Cologni G Uccelli MC Quiros-Roldan E Imberti L Airó P Pirovano S Patroni A Tirelli V Carosi G Immune correlates of virological response in HIV-positive patients after highly active antiretroviral therapy (HAART) Viral Immunol 2004 17 279 286 10.1089/0882824041310630 15279705

[B44] Moir S Buckner CM Ho J Wang W Chen J Waldner AJ Posada JG Kardava L O’Shea MA Kottilil S Chun TW Proschan MA Fauci AS B cells in early and chronic HIV infection: evidence for preservation of immune function associated with early initiation of antiretroviral therapy Blood 2010 116 5571 5579 10.1182/blood-2010-05-285528 20837780 PMC3031405

[B45] van Grevenynghe J Cubas RA Noto A DaFonseca S He Z Peretz Y Filali-Mouhim A Dupuy FP Procopio FA Chomont N Balderas RS Said EA Boulassel MR Tremblay CL Routy JP Sékaly RP Haddad EK Loss of memory B cells during chronic HIV infection is driven by Foxo3a- and TRAIL-mediated apoptosis J Clin Invest 2011 121 3877 3888 10.1172/JCI59211 21926463 PMC3195482

[B46] Hodge JN Srinivasula S Hu Z Read SW Porter BO Kim I Mican JM Paik C Degrange P Di Mascio M Sereti I Decreases in IL-7 levels during antiretroviral treatment of HIV infection suggest a primary mechanism of receptor-mediated clearance Blood 2011 118 3244 3253 10.1182/blood-2010-12-323600 21778338 PMC3179394

[B47] Sportès C Hakim FT Memon SA Zhang H Chua KS Brown MR Fleisher TA Krumlauf MC Babb RR Chow CK Fry TJ Engels J Buffet R Morre M Amato RJ Venzon DJ Korngold R Pecora A Gress RE Mackall CL Administration of rhIL-7 in humans increases in vivo TCR repertoire diversity by preferential expansion of naive T cell subsets J Exp Med 2008 205 1701 1714 10.1084/jem.20071681 18573906 PMC2442646

