# Supplementary material for: Effects of combined antiretroviral therapy on B- and T-cell release from production sites in long-term treated HIV-1+ patients
Source: J Transl Med. 2012 May 16;10:94. doi: 10.1186/1479-5876-10-94 (PMC3481359; doi:10.1186/1479-5876-10-94)
Supplement: Additional file 1 — Contains Figure S1, S2, and S3 (legend and artwork). [file 1479-5876-10-94-S1.pdf]

Additional File 1

Figure S1

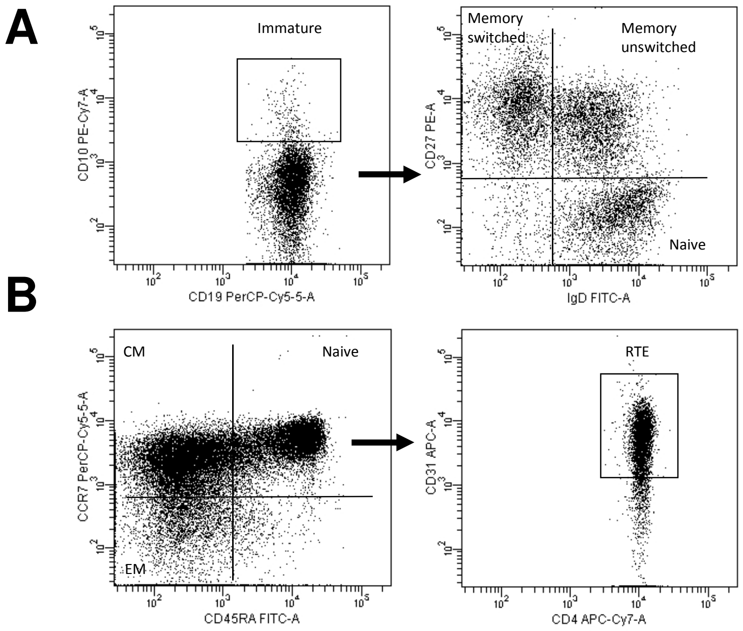

Gating strategy for the identification of B- and T-cell subsets. (A) For B-cell subsets characterization, fresh blood cells were stained with mAbs specific for CD19, CD10, CD27 and IgD. CD19$^+$ cells were first gated on lymphocytes and then analyzed for the expression of the other surface antigens. Immature B cells were defined as CD19$^+$CD10$^+$ lymphocytes; naive mature, memory unswitched, and memory switched B cells were CD19$^+$CD10$^-$IgD$^+$CD27$^-$, CD19$^+$CD10$^-$IgD$^+$CD27$^+$, and CD19$^+$CD10$^-$IgD$^-$CD27$^+$ lymphocytes, respectively.
(B) For T-cell subsets identification, mAbs for CD4, CD45RA, CCR7 and CD31 markers were used. CD4$^+$ cells were first gated on lymphocytes and then analyzed for the expression of the other surface antigens. Naive T cells were identified as CD4$^+$CD45RA$^+$CCR7$^+$ lymphocytes, RTE were naïve T cells expressing CD31$^+$ cells and T_{CM} and T_{EM} were CD4$^+$CD45RA$^-$CCR7$^+$ and CD4$^+$CD45RA$^-$CCR7$^-$ lymphocytes, respectively.

Figure S2

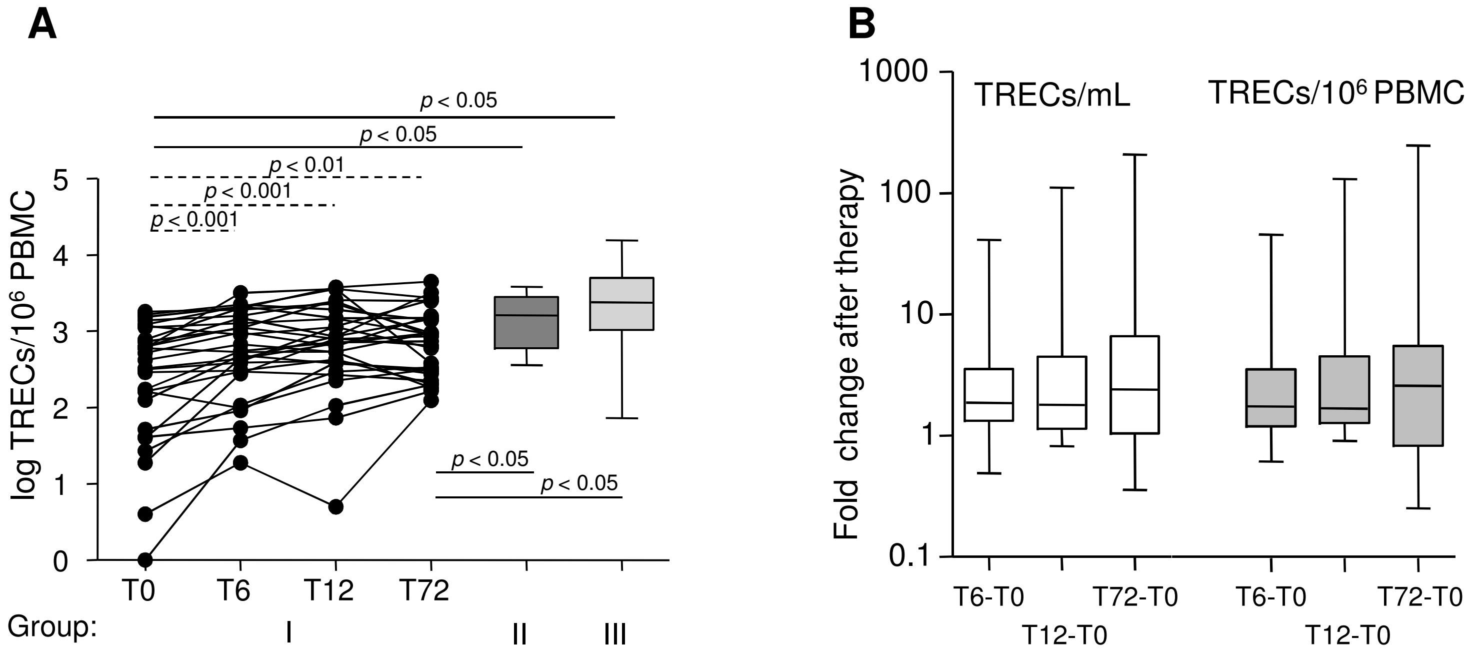

Comparison between therapy-induced changes of TRECs/10^6 PBMC and TRECs/mL measures.

(A) Measure of TRECs/10^6 PBMC in the 36 HIV-1^+ patients that initiated cART and were thereafter followed up for 72 months (group I, from T0 to T72), in the 22 HIV-1^+ patients that did not need therapy (group II, dark gray box), and in 72 age- and gender-matched uninfected subjects (group III, light gray box). Values found in individual patients are shown as filled circles, and dashed significance lines indicate the results of the longitudinal analysis, while solid significance lines indicate comparisons with the other groups.

(B) Fold change increase of TRECs/mL (clear boxes) and TRECs/10^6 PBMC (gray boxes) from T0 to T6, T12 and T72 in patients of group I.

Figure S3

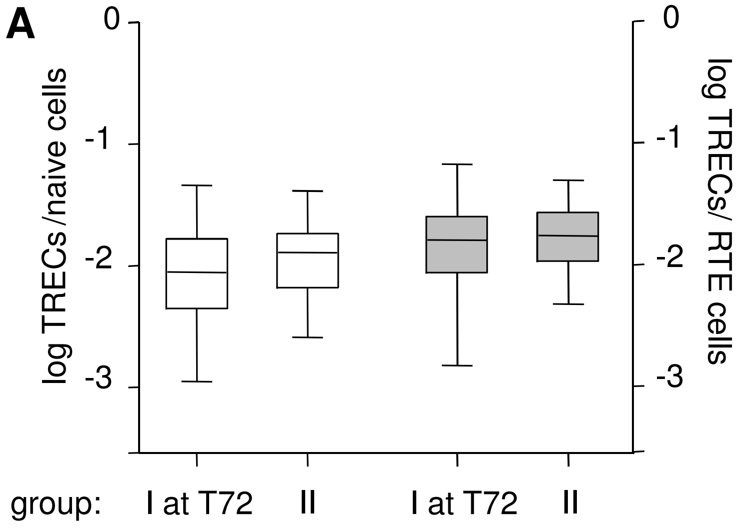

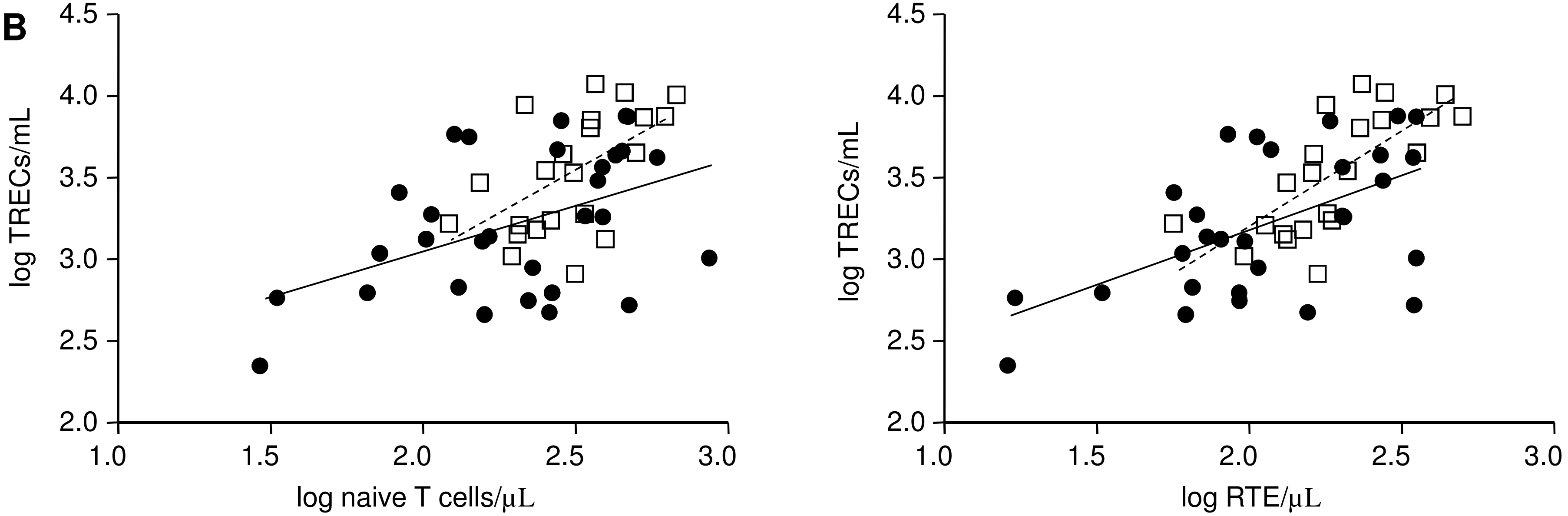

Evaluation of TRECs per naive T-cell and of TRECs per RTE density.

(A) Ratio of TRECs to naive T cells (clear boxes) and RTE cells (gray boxes) in samples of patients of group I at T72 (long-term treated) and of group II (untreated). No statistically significant differences were found with the t-test.

(B) Correlation between TRECs/mL and naive T cells or RTE/μL in patients of group I at T72 (black dots, solid line) and of patients of group II (clear squares, dashed line). Lines, obtained by linear regression, do not have a significantly different slope.
